# Effect of Intermittent Positive Pressure Ventilation on Depth of Anaesthesia during and after Isoflurane Anaesthesia in Sulphur-Crested Cockatoos (*Cacatua galerita galerita*)

**DOI:** 10.1155/2014/250523

**Published:** 2014-01-21

**Authors:** Saul Chemonges

**Affiliations:** Critical Care Research Group Laboratory, The Prince Charles Hospital, The University of Queensland, 3rd Floor, Clinical Sciences Building, Rode Road, Chermside, QLD 4032, Australia

## Abstract

This study aimed to determine the effect of intermittent positive pressure ventilation (IPPV) on the depth of inhalation anaesthesia in parrots. Anaesthesia was induced with 3.0% isoflurane in six Sulphur-crested Cockatoos (*Cacatua galerita galerita*) and maintained using either 1.5% or 3.0% during spontaneous ventilation (SV) or IPPV at 6 (IPPV-6) or 12 (IPPV-12) breaths per minute. The time taken for the appearance of somatic reflexes and the return of SV after IPPV was recorded. During recovery, the body jerk, beak, eye, and shivering reflexes appeared after 126 ± 27 s, 133 ± 26 s, 165 ± 34 s, and 165 ± 44 s, respectively. All cockatoos developed apnoea after IPPV-12 and only some did after IPPV-6. Return of SV after IPPV-12 was delayed compared to IPPV-6. Recovery times after the SV runs were significantly different between 1.5% and 3.0% isoflurane anaesthesia. Similarly, after IPPV, the recovery times were significantly different between 1.5% and 3.0% isoflurane anaesthesia. Recovery times after 3.0% inhaled isoflurane were longer than those of 1.5% inhaled isoflurane. In conclusion, cockatoos recovering from isoflurane anaesthesia are likely to exhibit body jerk, beak, eye, and shivering reflexes in that order. IPPV increases the depth of anaesthesia in a rate and dose-related manner and prolongs recovery.

## 1. Introduction

Isoflurane continues to be a popular anaesthetic agent for birds [1–3], due to its relative safety and effectiveness [4], and changes in the depth of anaesthesia and recovery can be easily and quickly controlled [5, 6]. Its faster induction and recovery, relative sparing effect on cardiovascular function and cerebral blood flow autoregulation, and negligible metabolism make isoflurane useful in the anaesthetic management of debilitated, aged, or exotic veterinary patients [3, 7]. Most of it is eliminated via the lungs with only a minute fraction metabolised in the liver [8–10]. Other anaesthetic inhalation agents such as sevoflurane [5, 6, 8, 9, 11–20] and desflurane [18] may have slight advantages over isoflurane; however, associated costs are considerable which limits their widespread use in veterinary practice.

The basic principles of anaesthetic management that govern mammalian anaesthesia also apply to birds, although specific anatomical and physiological differences must be considered [4, 9, 10, 21]. Because of the anatomy and structure of the avian respiratory system, even healthy birds may not be properly oxygenated when anaesthetised and placed in dorsal recumbence, so IPPV is recommended [10, 22]. Data on the effects of IPPV in birds is still relatively scarce compared to mammals [10]. In addition, the effects of isoflurane in cockatoos have been scantly documented. A study of isoflurane in anaesthetised Hispaniolan Amazon parrots did not notice any differences between IPPV and spontaneous ventilation [13]. One other relevant study on African grey parrots was documented on capnography during IPPV [23]. The aim of this study was to determine if IPPV increases the depth of anaesthesia in isoflurane anaesthetised cockatoos.

## 2. Materials and Methods

### 2.1. Anaesthetic Induction

The methods of this experiment have previously been described in related studies [3]. In brief, six Sulphur-crested Cockatoos (*Cacatua galerita galerita*) were used with approval from the University of Queensland Animal Ethics Committee (Permit no. CAS/118/98/D) and permission from the Australian Department of Environment (Permit no. E4/000229/98/SAA). Experiments were conducted in strict adherence to the Australian code of practice for the care and use of animals for scientific purposes.

The study was conducted in a room heated to 28°C. Anaesthesia was induced by masking using an Ayre's T-piece and 3.0% isoflurane (Forthane, Abbott, Australia). Thereafter, the birds were connected to a circle breathing system (CIG Midget 3, Australia) fitted with modified 10 mm internal diameter hoses, using either 1.5% or 3.0% isoflurane. The oxygen flow rate was set at 0.2 L/min. After allowing the anaesthesia to stabilise for 5 min, zero time was recorded. Anaesthetised birds were covered with bubble plastic to prevent heat loss and kept warm using an electric heating pad (Breville, China) maintained between 40° and 45°C.

The right thigh of the bird was deplumed and a paediatric digital blood pressure inflatable cuff (Hokanson, WA, USA) was placed around the proximal third of the tibiotarsal region. A Doppler probe was wetted with a conductive gel (SS250C, SEAL Systems, Australia) and secured distally to the inflatable cuff over the medial, midtibiotarsal region with a cohesive flexible bandage (Co-Flex, Andover, USA) to measure blood pressure (BP) by an ultrasonic Doppler flow detector (811-b, USA). The heart rate (HR) was determined by counting the pulse sound amplified by the partially inflated cuff of the Doppler flow detector. Cloacal temperature (*T*) was read from a standard clinical mercury thermometer. The respiratory rate (RR) was determined by counting abdominal movements during spontaneous respiration.

A 23-gauge indwelling IV catheter was placed and secured into the left jugular vein. Immediately before a blood sample was collected, the catheter was flushed by drawing blood into a separate 1 mL syringe and then back into the vein five times. Blood (1.5 mL) was collected into a heparinized syringe at times 0, 30, and 60 min. Approximately 1 mL of blood was put in a serum separator tube (Vacutainer, UK) and centrifuged for 3 min at 2,500 revolutions/min before being submitted to the laboratory for measurement of plasma electrolytes. The heparinized syringe containing 0.5 mL of blood was capped, placed in ice, and blood gas and acid-based parameters were measured within 30 min using a blood gas machine (Chiron Diagnostics, Australia).

Each bird was subjected to three different anaesthetic experimental sets in a crossover pattern allowing a recovery and washout period of three days between each set of the experiments. In one experimental set, the bird was spontaneously ventilated (SV). The other two sets of experiments involved alternation between SV and two rates of intermittent positive pressure ventilation (IPPV-6 and IPPV-12).

### 2.2. Control Experiment for Baseline Parameters

During 3.0% of isoflurane anaesthesia, five consecutive 10 min periods of SV were studied with the birds connected to the T-piece. BP, RR, HR and *T* were recorded at the start, during, and at the end of each period. At the end of each period, the bird was then disconnected from the T-piece and the endotracheal tube connector was tapped with the author's index finger every 5 s. The body jerk, beak, eye, shivering, and righting reflexes were monitored and the time taken for these reflexes to appear was recorded. When the righting reflex appeared, the bird was reconnected to the anaesthetic machine and anaesthesia was allowed to stabilise for 5 min before the procedure was repeated 4 more times.

### 2.3. Determination of the Depth of Anaesthesia

To determine the depth of anaesthesia during SV and IPPV, the procedure used in the SV control experiment was repeated 3 times with the birds inhaling 1.5% isoflurane. After the first SV run, the bird was reconnected to the circle absorber with low volume hoses of 10 mm internal diameter for 5 min, and then IPPV-6 using 4 mmH_2_O was administered for 10 min with a Campbell respirator (ULCO Pty, NSW). The bird was disconnected from the anaesthetic machine and the time taken for the return of spontaneous respiration and reflexes was recorded. After the second SV anaesthesia run, IPPV rate was changed to IPPV-12. This procedure was also repeated in all the birds using 3.0% inhaled isoflurane.

After each set of experiments, the intravenous catheter was removed, the endotracheal tube cuff deflated, and the bird was disconnected from the anaesthetic machine. The bird was wrapped in a towel, placed in a bird cage, and monitored until it recovered fully from the anaesthetic.

### 2.4. Statistics

Data were tabulated and processed using Microsoft Excel spread sheet. Values were expressed as mean ± SD and differences at *P* < 0.05 were considered significant.

## 3. Results

### 3.1. Baseline Parameters during SV

Mean values (± SD) for HR, BP, RR, and *T* did not significantly change during the SV study (Table 1). Not all the reflexes monitored were observed in all the birds, except for the definitive righting reflex. The righting reflex appeared when the bird awoke from anaesthesia and the time of appearance progressively increased significantly (*P* < 0.01) with repeated anaesthesia. In birds that exhibited shivering, there was a progressive shortening in the time at which the reflex appeared; these time periods were significantly different (*P* < 0.05) between the individual SV runs. The eye reflex occurred immediately prior to the righting reflex.

The results from venous blood gas analysis during SV experiments are displayed in Table 2. Except for pH, base excess, and O_2_ saturation, there was a significant increase (*P* < 0.05) in pCO_2_ and pO_2_.

### 3.2. Depth of Anaesthesia during SV and IPPV

During the SV runs with 1.5% inhaled isoflurane, the HR and RR decreased significantly (*P* < 0.05), while BP and *T* remained relatively stable (Table 3). During IPPV-6, BP was significantly different (*P* < 0.05) from the SV runs. HR, BP, and *T* for both IPPV runs were not significantly different from those of SV runs. During SV runs with 3.0% inhaled isoflurane, HR increased significantly (*P* < 0.05), while the RR decreased. BP and *T* remained relatively constant (Table 3). Insignificant decreases were noted in HR and BP during IPPV with 3.0% inhaled isoflurane.

During SV and IPPV runs while inhaling 3.0% isoflurane, there were noticeable increases in venous blood pH initially accompanied with a significant decrease in pCO_2_, whilst pO_2_ and BE remained stable (Table 4). Data for SV and IPPV while inhaling 1.5% isoflurane is not available.

The body jerk, shivering, beak, and eye reflexes were not observed in all the birds after SV and IPPV (Table 5). For 1.5% inspired isoflurane in the birds where the body jerk reflex appeared, there was a progressive and significant (*P* < 0.05) delay in the appearance of the reflex after the SV runs. The time for the appearance of the body jerk reflex for IPPV-6 was shorter than that of IPPV-12. The shivering reflex appeared sooner following IPPV-6 than following IPPV-12. The time for the appearance of the beak reflex after the SV runs was relatively constant; however, it changed significantly during IPPV. The beak reflex was further delayed after IPPV-12 than after IPPV-6. There was a progressive significant increase (*P* < 0.05) in the time of appearance of the eye reflex after the SV runs.

After 3.0% inspired isoflurane, there was a significant increase (*P* < 0.05) in the time of appearance of the body jerk reflex after the SV and IPPV runs (Table 6). This reflex was delayed significantly (*P* < 0.05) after IPPV-12 compared to that of IPPV-6. The shivering reflex was observed only in one bird during the third SV run. The appearance of the beak reflex was delayed more significantly (*P* < 0.05) after IPPV-12 than after IPPV-6. There was a significant difference (*P* < 0.05) in the appearance of the eye reflex during both IPPV and SV runs. Overall, the time taken for the return of all the reflexes increased for both SV and IPPV. The appearance of reflexes was delayed more significantly (*P* < 0.05) after IPPV than after SV runs. The duration of return of the reflexes after IPPV-12 runs was significantly (*P* < 0.05) higher than those of IPPV-6.

The recovery time progressively increased and differed significantly within the SV (*P* < 0.01) and IPPV (*P* < 0.001) runs after 1.5% and 3.0% inhaled isoflurane (Table 6). Recovery times during the SV runs were significantly different (*P* < 0.05) between birds that had inhaled 1.5% and 3.0% isoflurane. Also, during the IPPV runs, the recovery times were significantly different (*P* < 0.001) between 1.5% and 3.0% isoflurane anaesthesia.

Some birds developed apnoea after IPPV-6, but all the birds developed apnoea after IPPV-12 (Table 7). The time taken for the return of spontaneous breathing in apnoeic birds was significantly different (*P* ≪ 0.001) between IPPV-6 and IPPV-12, after either 1.5% or 3.0% isoflurane anaesthesia. Return of spontaneous breathing occurred sooner after IPPV-6 than after IPPV-12.

## 4. Discussion

The goal in anaesthesia is to maintain the lowest possible level of anaesthesia to achieve the necessary restrain, minimise stress, and induce muscle relaxation and to mitigate pain nociception. Anaesthetic planes in birds are difficult to assess from outward signs [24]; however, the depth of anaesthesia can be estimated by combining information obtained objectively from HR, RR, and reflexes [9, 14, 15, 17]. The signs of anaesthetic depth depend upon the evaluation of muscular tone and muscular reflexes [24]. The use of HR, RR, BP, and reflexes is well recognised in veterinary anaesthesia. Signs of anaesthetic depth vary among species and anaesthetic drugs [24]. In birds, the published and commonly monitored reflexes published include the palpebral, pedal, cere, withdrawal, pain on feather pluck, pain, and muscle tone [1, 9, 14, 15, 17, 21]. Light planes of anaesthesia are more associated with the presence of more reflexes. In the present study, the reflexes monitored were descriptive surrogates of the commonly known monitored reflexes appearing as the plane of anaesthesia lightened during recovery [10]. These were body jerk (emergence of muscle tone), beak (emergence of jaw tone), eye (cere), shivering (wing tone), and the righting reflexes (consciousness). Other studies in birds have used toe pinch, corneal reflexes, and wing tone [5, 6, 9, 15, 16, 25]. The signs of anaesthetic depth, however, vary from individual to individual and from moment to moment during a single anaesthetic episode, because of adjunctive events such as surgical stimulation and body temperature [24]. There were interindividual variations in the appearance of the reflexes for monitoring the depth of anaesthesia in this study. The righting reflex signified that the bird was awake and was the only consistent one in all the birds. All evaluations of anaesthetic depth based on the reflexes were interpreted as single point-in-time measurement, which underscores reflex variability concerns previously raised by other investigators [13, 24].

The isoflurane concentrations used in this study were 1.5% and 3.0%; other studies have used lower concentrations (1.34 ± 0.14%) of isoflurane [25]. In the present study, 3.0% inspired isoflurane produced induction and more stable anaesthesia than 1.5% during SV. Some birds tended to wake up during SV and 1.5% isoflurane anaesthesia within minutes after changeover to the circle absorber. Birds did not wake during IPPV and 1.5% isoflurane, suggesting that the use of a continuous delivery of isoflurane anaesthetic rather than relying on spontaneous ventilation is necessary to prevent wakening, especially during the induction phase with 1.5% isoflurane. Although nonrebreathing semiopen anaesthesia circuits have been recommended for inhalant delivery in earlier studies [4], a rebreathing circuit with low volume hoses of 10 mm internal diameter and a relatively high oxygen flow rate was satisfactorily used in this study.

The efficiency of the avian respiratory system accounts for the rapid induction, changes in depth of anaesthesia, and speed of recovery when isoflurane is used [6, 9, 17, 18, 21]. Recovery from isoflurane is primarily due to the elimination of the gas by the lungs [5, 18, 21, 25]. Because of their small size and high body surface to volume ratio and the use of high oxygen flow rates, hypothermia develops quickly during anaesthesia in birds [4, 21]. For this reason, a heating pad and the bubble plastic cover were necessary to maintain the normal body temperature of the birds during the experiments.

Peak positive pressures of 15–20 cmH_2_O have been used in anaesthetising waterfowls. Other studies suggest that the airway pressure during IPPV in birds should not exceed 12 to 20 cmH_2_O to prevent barotrauma to the air sacs [4, 12]. In this study a peak pressure of 4 cmH_2_O was used to ventilate the cockatoos and was sufficient to raise the birds' abdomen considerably and yielded satisfactory ventilation as supported by venous blood gas analysis. Contrary to earlier reports [4], IPPV pressures much greater than 4 cmH_2_O appear to be excessive and more comparative studies are indicated. As observed in earlier studies [3], IPPV resulted in better ventilation leading to mild hypocapnoea. Whilst arterial blood samples would have been desirable for arterial blood gas analysis, venous blood samples as used in this study within known limitations are an acceptable surrogate for ABG analysis [26–29]. The observed decrease in pO_2_ over time during IPPV and the tendency for base excess to shift towards a more positive value may suggest a respiratory inefficiency due to lung injury. This potentially undesirable effect of IPPV was not observed during SV and can only be confirmed if arterial pO_2_ is known so as to determine the arterial oxygen tension to inspired fraction of oxygen (PaO_2_ : FiO_2_) ratio.

The minor changes in HR during repeated anaesthesia episodes suggest that isoflurane has little influence on BP. This observation is consistent with findings in other studies [9, 15]. Isoflurane could have counteracted other vasodilatory mechanisms associated with it, contributing to the maintenance of vascular tone during anaesthesia [11]. This probably explains why BP remained steady during isoflurane anaesthesia in the cockatoos. Studies in dogs suggest that there is a reduction of cardiac work induced by isoflurane, which plays a role in myocardial protection [30], but it is not known if this applies to birds.

Several factors can cause respiratory depression in anaesthetised birds [25, 31]. Hypoventilation can be due to anaesthetic-induced depression of the central nervous system, relaxation of the muscles of respiration, and effects on central and peripheral chemoreceptors [13]. The position of the bird during anaesthesia can also affect ventilation significantly contributing to hypoventilation [2]. Ventilation is reduced in birds that are placed on their backs [1, 31], and exposure of the larynx and upper trachea to inhaled cold air also slows respiration [1]. Compared with mammals, the respiratory function in birds may be more sensitive to the effects of inhalant anaesthetics because of their effect on the unique carbon dioxide-sensitive intrapulmonary chemoreceptors [31]. High fractions of inspired oxygen may also contribute to hypoventilation [32], possibly by depressing oxygen-sensitive chemoreceptors that are sensitive to partial pressure of carbon dioxide (pCO_2_) and many similar chemoreceptors [33]. The intrapulmonary chemoreceptors found in birds, however, are not mechanoreceptors; they are sensitive to carbon dioxide and insensitive to hypoxia [34, 35]. There could have been a decreased responsiveness to increased blood CO_2_ tension, as this occurs during isoflurane anaesthesia [24]. Therefore, without sufficient CO_2_, hypoventilation is expected to occur.

The monitored reflexes that appeared during recovery from isoflurane anaesthesia suggest that there is a dose-related delay in recovery. Reflexes appeared sooner after 1.5% isoflurane anaesthesia than after 3.0%. Intermittent positive pressure ventilation delayed the appearance of the reflexes more than SV. Also, the IPPV rate influenced the appearance of the reflexes; they appeared earlier during IPPV at 6 breaths/min than at 12 breaths/min. The delay in the appearance of reflexes during recovery at a given inhaled concentration and ventilation type suggests a probable residual effect from the previous anaesthetic episode. A dose-related prolongation of recovery from anaesthesia occurred, that was well marked during IPPV, and increased with a higher IPPV rate. The higher the IPPV rate, the deeper the anaesthesia and the longer it took for recovery from anaesthesia. These observations contrast those of Pettifer and colleagues [13].

During recovery from isoflurane anaesthesia, the visible reflexes to be seen, if they occurred in the cockatoos, followed a sequence. The order of appearance of the reflexes was body jerk, beak, eye, shivering, and righting reflex. The eye, body, beak, and shivering reflexes, in descending order, were most likely to be seen in a greater proportion of cockatoos.

The decrease in HR and RR and the slight decrease in BP in the control experiment suggest a progressive depression of the respiratory and cardiovascular system. Again, this was probably due to repeated episodes of isoflurane anaesthesia within short intervals. This depression would probably be unnoticeable if the time interval between the runs was increased to allow the birds' physiological status to return to normal by getting rid of the residual effects of the previous anaesthetic episode.

Although the BP was higher during 3.0% inspired isoflurane and lower during 1.5% for both SV and IPPV, it was still within the known avian BP range of 110 to 220 mmHg [7, 13]. In agreement with this, a study reported that mean arterial BP in birds is higher during IPPV than SV [25]. Other studies have also indicated that the arterial BP values recorded during maintenance of general anaesthesia in birds tend to be higher than those recorded for most mammalian species [16, 36]. From these observations, it is evident that at normal body temperature during isoflurane anaesthesia in cockatoos, cardiovascular parameters are influenced more by isoflurane concentration than by the mode of ventilation.

Apnoea was observed in all birds after IPPV-12 and in some birds after IPPV-6. This was probably because IPPV (and not SV) overcame the apnoeic threshold in the birds that became apnoeic. The apnoeic threshold corresponds to the arterial pCO_2_ where the spontaneous ventilatory effort ceases [37]. It occurs when the arterial pCO_2_ is reduced by 5 to 9 mmHg by voluntary hyperventilation or by artificial ventilation. The gap between resting arterial pCO_2_ level and apnoeic threshold is relatively constant, irrespective of the anaesthetic depth [37]. In this study the venous pCO_2_ decreased by 4 mmHg; therefore, it is possible that the apnoeic threshold was not reached in birds that did not develop apnoea after IPPV-6. Also, birds have been reported to be very sensitive to CO_2_ concentrations, and depletion of CO_2_ causes acute apnoea [25]. IPPV-12 possibly depleted stimulatory actions of arterial CO_2_ resulting in apnoea in all the birds. Birds that became apnoeic after subjection to IPPV-6 spontaneously ventilated earlier than those subjected to IPPV-12. The prolongation of return of SV at a higher dose of isoflurane and IPPV rate implied that there is a dose-related delay in return of spontaneous ventilation after apnoea. This suggests that the rate of ventilation and, to a lesser extent, isoflurane concentration influenced the return of spontaneous ventilation after apnoea. Therefore, from these observations, IPPV appears to increase the depth of anaesthesia in a rate and dose-related manner during isoflurane anaesthesia.

This study has some limitations regarding the interpretation of the results of IPPV-12. It did not take into account the influence of the length of anaesthesia on this run as it was done always last in the same anaesthesia episode as IPPV-6. A more desirable approach would have been to have different groups with a crossover pattern with IPPV-6 coming last in equal proportion in some experiments to exclude this influence. Logistical constraints prevented blood gas analysis for 1.5% inhaled isoflurane group of experiments. A more objective reflex score would have enhanced the statistical comparison of the results.

## 5. Conclusion

Isoflurane anaesthesia in cockatoos is rapidly induced and recovery is also rapid. Repetitive isoflurane anaesthesia in spontaneously ventilating cockatoos has little effect on BP and HR, and outcomes are similar for SV and IPPV although IPPV results in better cardiovascular stability than SV. Cockatoos recovering from isoflurane anaesthesia are likely to exhibit body jerk, beak, eye, shivering, and the righting reflex, in that chronological order of appearance. IPPV increases the depth of anaesthesia in a rate and dose-related manner, which in turn prolongs recovery. High rates of IPPV and higher doses of isoflurane cause apnoea, which in turn prolongs recovery from anaesthesia. IPPV provides an opportunity to use lower isoflurane concentrations to achieve a desirable level of anaesthesia.

## Figures and Tables

**Table 1:** Mean ± SD values for heart rate (HR), blood pressure (BP), respiratory rate (RR), temperature (*T*), and time taken for reflexes to appear in 6 sulphur-crested cockatoos (*Cacatua galerita galerita*) inhaling 3.0% isoflurane subjected to 5 identical runs of spontaneous ventilation (SV) anaesthesia.

SV run	HR (beats/min)	BP (mmHg)	RR (breaths/min)	*T* (°C)	Time for return of reflex (s)				
					Righting	Body jerk	Shivering	Beak reflex	Eye reflex
(i)	245 ± 58	186 ± 31	49 ± 35	40 ± 0.6	148 ± 40	108 ± 33 (3)	0	126 ± 33 (3)	170 ± 57 (2)
(ii)	247 ± 49	184 ± 35	43 ± 30	40 ± 0.6	163 ± 43	143 ± 55 (2)	186 ± 70 (2)	132 ± 33 (4)	172 ± 59 (3)
(iii)	227 ± 37	184 ± 30	39 ± 23	39 ± 0.4	160 ± 27	130 (1)	168 ± 45 (2)	145 ± 35 (2)	154 ± 14 (3)
(iv)	223 ± 39	183 ± 35	34 ± 19	39 ± 0.6	179 ± 35	135 ± 24 (4)	176 ± 56 (2)	129 ± 21 (4)	174 ± 16 (3)
(v)	229 ± 32	182 ± 37	36 ± 25	39 ± 0.7	188 ± 97	120 ± 9 (3)	132 ± 4 (2)	134 ± 30	158 ± 86 (3)

Numbers in parentheses indicate the number of birds where the reflex was observed.

**Table 2:** Mean ± SD values for venous blood hydrogen ion concentration (pH), carbon dioxide partial pressure (pCO_2_), oxygen partial pressure (pO_2_), bicarbonate (HCO_3_^−^), base excess (BE), and haemoglobin oxygen saturation (O_2_ Sat) during spontaneous ventilation (SV) while inhaling 3.0% isoflurane in 100% oxygen in sulphur-crested cockatoos (*Cacatua galerita galerita*).

Time (min)	pH	pCO_2_ (mmHg)	pO_2_ (mmHg)	HCO_3_^−^ (mmol/L)	BE (mmol/L)	O_2_ sat. (%)
0	7.4 ± 0	38.3 ± 5	101.8 ± 12	24.3 ± 1.6	0.3 ± 1	98 ± 0.6
30	7.4 ± 0	52.4 ± 7	114.8 ± 9	29.8 ± 1.7	11.2 ± 2	98 ± 0.2
60	7.4 ± 0	61.8 ± 14	120.9 ± 56	31.5 ± 3.4	3.7 ± 1.8	99 ± 0.4

Adapted and modified with permission from Chemonges, “Effect of intermittent positive pressure ventilation (IPPV) on acid-base balance and plasma electrolytes during isoflurane anaesthesia in sulphur-crested cockatoos (*Cacatua galerita galerita*),” [3].

**Table 3:** Mean ± SD values for heart rate (HR), respiratory rate (RR), blood pressure (BP), and temperature (*T*), measured during either spontaneous ventilation (SV) or intermittent positive pressure ventilation (IPPV) in 6 sulphur-crested cockatoos (*Cacatua galerita galerita*) during 1.5% and 3.0% isoflurane anaesthesia.

Ventilation	HR (beats/min)		BP (mmHg)		RR (breaths/min)		*T* (°C)	
	1.5%	3.0%	1.5%	3.0%	1.5%	3.0%	1.5%	3.0%
SV	253 ± 39	208 ± 94	175 ± 26	194 ± 39	67 ± 43	64 ± 42	40 ± 0.6	40.6 ± 0.6
IPPV-6	175 ± 29	243 ± 57	170 ± 28	188 ± 43	—	—	39.8 ± 0.6	40.3 ± 0.3
SV	165 ± 54	243 ± 22	171 ± 34	184 ± 37	37 ± 24	54 ± 54	40 ± 0.4	39.9 ± 0.2
IPPV-12	183 ± 40	231 ± 39	156 ± 30	178 ± 34	—	—	39.4 ± 0.5	39.5 ± 0.2
SV	177 ± 40	251 ± 21	169 ± 26	187 ± 37	34 ± 22	46 ± 34	39 ± 0.4	39.2 ± 0.4

**Table 4:** Mean ± SD values for venous hydrogen ion concentration (pH), carbon dioxide partial pressure (pCO_2_), oxygen partial pressure (pO_2_), bicarbonate (HCO_3_^−^), base excess (BE), and haemoglobin oxygen saturation (O_2_ Sat) during intermittent positive pressure ventilation (IPPV) with peak inspiratory pressure of 4 cmH_2_O at 12 respirations per minute inhaling 3.0% isoflurane in 100% oxygen in 6 sulphur-crested cockatoos (*Cacatua galerita galerita*).

Time (min)	pH	pCO_2_ (mmHg)	pO_2_ (mmHg)	HCO_3_^−^ (mmol/L)	BE (mmol/L)	O_2_ sat. (%)
0	7.4 ± 0	37.3 ± 6	110 ± 24	22 ± 3	−2.8 ± 3	98 ± 1
30	7.5 ± 0	33.1 ± 3	72 ± 9	26 ± 2	3 ± 2	96 ± 1
60	7.5 ± 0	34.3 ± 4	82 ± 12	26 ± 2	3.6 ± 2	97 ± 1

Adapted and modified with permission from Chemonges, “Effect of intermittent positive pressure ventilation (IPPV) on acid-base balance and plasma electrolytes during isoflurane anaesthesia in sulphur-crested cockatoos (*Cacatua  galerita  galerita*),” [3].

**Table 5:** Mean ± SD values for the duration it took for the appearance of the body jerk, shivering, beak, and eye reflexes measured repeatedly after either SV or IPPV in 6 sulphur-crested cockatoos (*Cacatua  galerita  galerita*) after 1.5% and 3.0% isoflurane anaesthesia.

	Time for the appearance of reflex (s)							
Ventilation	Body jerk		Shivering		Beak reflex		Eye reflex	
	1.5%	3.0%	1.5%	3.0%	1.5%	3.0%	1.5%	3.0%
SV	59 (1)	58 (1)	111 ± 7 (3)	−(0)	83 ± 35 (3)	−(0)	49 ± 1 (2)	136 ± 15 (2)
IPPV-6	70 ± 12 (3)	139 ± 46 (3)	96 ± 38 (3)	−(0)	85 ± 17 (3)	191 ± 44	121 ± 13 (3)	203 ± 33 (2)
SV	86 ± 10 (3)	148 ± 21 (2)	116 ± 40 (4)	−(0)	80 ± 20	176 ± 73 (3)	95 ± 50 (3)	164 ± 46
IPPV-12	135 ± 30 (3)	271 ± 132 (4)	166 ± 17 (4)	−(0)	124 ± 52 (4)	262 ± 112	145 ± 49 (3)	340 ± 80 (2)
SV	106 ± 38 (3)	131 ± 59 (3)	110 ± 33 (4)	402 (1)	93 ± 17	195 ± 121 (5)	111 ± 12 (3)	269 ± 154

Numbers in parentheses indicate the number of birds in which the reflex was observed.

**Table 6:** Mean ± SD for time it took for the righting (recovery) reflex to appear after spontaneous (SV) or intermittent positive pressure ventilation (IPPV) in 6 sulphur-crested cockatoos (*Cacatua galerita galerita*) after 1.5% or 3.0% isoflurane anaesthesia.

Respiration type	Appearance of righting reflex (s)	
	1.5 % isoflurane	3.0% isoflurane
SV	96 ± 28	135 ± 45
IPPV-6	114 ± 46	184 ± 37
SV	128 ± 32	182 ± 52
IPPV-12	195 ± 26	371 ± 132
SV	183 ± 87	228 ± 104

**Table 7:** Mean time ± SD for the return of spontaneous breathing in 6 sulphur-crested cockatoos (*Cacatua  galerita galerita*) during recovery from 1.5% and 3.0% isoflurane anaesthesia after intermittent positive pressure ventilation (IPPV).

IPPV rate (breaths/minute)	Time for return of spontaneous ventilation (s)	
	1.5% isoflurane	3.0% isoflurane
6	11 ± 8 (3)	17 ± 3 (3)
12	38 ± 22	53 ± 23

Numbers in parentheses indicates the number of birds that developed apnoea.
